# An update on molecular cat allergens: Fel d 1 and what else? Chapter 1: Fel d 1, the major cat allergen

**DOI:** 10.1186/s13223-018-0239-8

**Published:** 2018-04-10

**Authors:** B. Bonnet, K. Messaoudi, F. Jacomet, E. Michaud, J. L. Fauquert, D. Caillaud, B. Evrard

**Affiliations:** 1 Laboratoire d’Immunologie, ECREIN, UMR1019 Unité de Nutrition Humaine, Université Clermont Auvergne, 63000 Clermont-Ferrand, France; 2 Service d’Immunologie, CHU Clermont-Ferrand, 63000 Clermont-Ferrand, France; 3 Laboratoire de Biochimie, CHU Angers, Angers, France; 4 Laboratoire d’Immunologie, CHU Poitiers, Poitiers, France; 5 Service de Pédiatrie, CHU Clermont-Ferrand, 63000 Clermont-Ferrand, France; 6 Service de Pneumologie, CHU Clermont-Ferrand, 63000 Clermont-Ferrand, France

**Keywords:** Cat allergy, Fel d 1, CRD, Immunotherapy

## Abstract

**Background:**

Cats are the major source of indoor inhalant allergens after house dust mites. The global incidence of cat allergies is rising sharply, posing a major public health problem. Ten cat allergens have been identified. The major allergen responsible for symptoms is Fel d 1, a secretoglobin and not a lipocalin, making the cat a special case among mammals.

**Main body:**

Given its clinical predominance, it is essential to have a good knowledge of this allergenic fraction, including its basic structure, to understand the new exciting diagnostic and therapeutic applications currently in development. The recent arrival of the component-resolved diagnosis, which uses molecular allergens, represents a unique opportunity to improve our understanding of the disease. Recombinant Fel d 1 is now available for in vitro diagnosis by the anti-Fel d 1 specific IgE assay. The first part of the review will seek to describe the recent advances related to Fel d 1 in terms of positive diagnosis and assessment of disease severity. In daily practice, anti-Fel d 1 IgE tend to replace those directed against the overall extract but is this attitude justified? We will look at the most recent arguments to try to answer this question. In parallel, a second revolution is taking place thanks to molecular engineering, which has allowed the development of various forms of recombinant Fel d 1 and which seeks to modify the immunomodulatory properties of the molecule and thus the clinical history of the disease via various modalities of anti-Fel d 1-specific immunotherapy. We will endeavor to give a clear and practical overview of all these trends.

## Background

Worldwide, the domestic cat, *Felis domesticus*, is one of the most frequently encountered pets. It is a major source of allergens in the indoor environment and is placed in second position after dust mites for its involvement in the incidence of allergic respiratory diseases. In Western countries, the prevalence of sensitization to allergens of cat has increased dramatically to 10–30% in the general population [1]. A significant proportion of atopic subjects (about 20–40%) are sensitized to cat allergens [2, 3]. The severity of induced symptoms varies widely and cat allergy is thus a main risk factor of both rhinitis and asthma, including severe asthma, which can develop into a life-threatening condition.

Cat allergens have significant allergenicity. They are also numerous and cat allergen extracts are therefore a multi-allergenic source. Historically, 10 allergens recognized by specific IgE have been identified in studies of extracts from fur, saliva, serum and urine [4, 5]. Eight cat allergens have been registered to date in the WHO/IUIS allergen nomenclature (Fel d 1 to Feld d 8). The development of the component-resolved diagnosis (CRD), which uses molecular allergens produced by genetic engineering, offers new possibilities to improve the diagnosis and understanding of cat allergies [6]. The most important cat allergen in disease pathogenesis is, unlike in other mammals, a secretoglobin, called Fel d 1, and not a lipocalin [7]. Its predominance, shown by inhibition studies, is such that it is classically recognized as the major cat allergen, the only one whose clinical impact is essential [8].

The aim of this first chapter is to review the basic knowledge of Fel d 1 and to give an update on new clinical data, particularly the most recent clinical studies on the Fel d 1-based CRD of cat allergy and the various modalities of Fel d 1-specific immunotherapy.

## Main text

### Fel d 1, an uteroglobin-like protein

#### Molecular characteristics

**Fig. 1:**
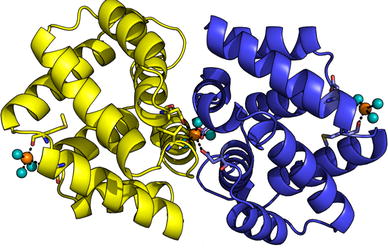
Fel d 1 crystallographic structure highlighting the location of the calcium ions. From Ligabue-Braun et al. [16] reprints in open access

Fel d 1 is a glycoprotein of about 35–38 kDa [9, 10]. It consists of two identical heterodimers, each of 18–19 kDa, linked noncovalently and eventually forming a tetramer [10]. Each dimer consists of two polypeptide chains, chain 1 and chain 2, covalently linked by three disulfide bridges and encoded by two different genes [11, 12]. Chain 1 (or α) consists of 70 amino acids and has a molecular weight of 8 kDa. This polypeptide has a marked structural identity with the rabbit lipophilin/secretoglobin (Ory c 3) and sequence homology with another member of the uteroglobin family, a protein of the human bronchial epithelial cells called Clara cell 10-kDa protein [11, 13]. Chain 2 (or β) is a glycoprotein of 10 kDa with *N*-oligosaccharides. It consists of 85, 90 or 92 amino acids [9]. Fel d 1 in its natural form is thought to be a mix of full and truncated forms of chain 2 [14]. The three-dimensional structure of Fel d 1 was determined, it is more complex than that of other allergens, with an internal cavity which could accommodate an endogenous ligand and two calcium external binding sites [9, 15, 16] (Fig. 1). Hence, the expression of recombinant Fel d 1 was more difficult to obtain than for other allergens. Each chain was first produced separately in simple systems using *Escherichia coli* (*E. coli*). However, to produce the full molecule rFel d 1, it was necessary to use a Baculovirus [14]. The advantage is that rFel d 1 is glycosylated (unlike products obtained via recombinant *E. coli*) and has a similar structure to that of the natural cat allergen nFel d 1 [14]. The epitopes of Fel d 1 are partially conformational because the amount of IgE reactivity directed against each of the two chains of Fel d 1 separately is far less than that of total IgE reactivity against the natural heterodimer [17, 18].

#### Biological function, family

Fel d 1 belongs to the family of secretoglobins or secretory globins [15]. The biological function of Fel d 1 is still unknown. It has been suggested that its role is to protect the skin, by homology with the uteroglobin whose function is to protect mucosa [19]. Other authors believe that Fel d 1 would rather have a role in the transport of lipid molecules, especially steroids, hormones or pheromones [20].

#### Epidemiology

Fel d 1 is a thermostable protein found in the saliva, anal glands, sebaceous glands, skin and fur of cats [11, 21, 22]. It is now recognized that the sebaceous glands, and not saliva, are the main production site [21–23].

All cats produce Fel d 1, but hormonal status modifies its production. For example, it has been shown that males produce more Fel d 1 than females [24]. In addition, castrated male cats produce less Fel d 1 than non-castrated males [25]. Not all cats shed Fel d 1 in the air at the same rate [26]. Production of Fel d 1 on the skin varies according to anatomical site and, for example, is much greater on the head than on the chest. The same distribution of Fel d 1 is found in the fur. The length of hair does not seem to affect the production of Fel d 1. Washing cats reduces the amount of Fel d 1 on the skin and fur but the effect does not last long as the amount of Fel d 1 returns to its original level in just 2 days [27, 28]. Similarly, the amount of Fel d 1 in ambient air is restored within 24 h [29]. Washing the cat is thus of little benefit.

In two large national surveys in the United States, Fel d 1 was detected, respectively, in 99.9 and 99.7% of American homes [30, 31]. Fel d 1 was found in the dust of sofas, carpets and beds in homes with cats, but also in homes without a cat [30]. High levels of Fel d 1 were also found in the classroom, in cars, the offices of allergists, and shopping centers [32–34]. Fel d 1 allergen is ubiquitous. It is likely that it spreads from the clothes of cat owners and may also spread from their hair.

60% of airborne Fel d 1 is carried by small particles, of which 75% are more than 5 microns in diameter and 25% less than 2.5 microns [35]. The immediate bronchial response to Fel d 1 appears to be located in the proximal airways. The concentration of Fel d 1 required to induce a positive bronchial response in subjects with intermittent asthma was 20 times less when the allergen was carried by large particles (10.3 μm) than when Fel d 1 was carried by small particles (1.4 μm) [36]. However, a more recent article suggests that exposure under natural conditions to cat allergens (and not just to Fel d 1) induces a more peripheral airway obstruction [37].

Fel d 1 is easily airborne and remains in the indoor environment but the relationship between antigenic load and onset of symptoms is not as clear as with mite-borne antigens [38]. There is still considerable difference of opinion on this subject. Several studies have reported a paradoxical effect of the presence of animals at home. While the presence of a cat in childhood seems to be a risk factor for sensitization and for developing asthma, children heavily exposed to a cat probably have a lower risk of developing a cat allergy [39–41]. Another large prospective study showed that living with a cat during childhood, especially during the first year of a child’s life, could be protective against allergic diseases [42]. Recent studies confirm these data, reporting that cat ownership during pregnancy and childhood in a large birth cohort (Avon Longitudinal Study of Parents and Children or ALSPAC) was consistently associated with a reduced risk of aeroallergen sensitization, wheezing and atopic asthma at the age of 7, but tended to be associated with an increased risk of non-atopic asthma [43, 44]. In another study, Carlsen’s team showed that the acquisition of a pet in early life did not appear to either increase or reduce the risk of asthma or allergic rhinitis symptoms in children aged 6–10 years [45]. Conversely, a recent French study (named PARIS) of 1860 infants reported that a cat entering the baby’s room in early life was strongly associated with aeroallergen sensitization (ORa 3.21, 95% CI 1.29–8.01), particularly against Fe l d 1 [46]. An interesting explanation of these contradictory results could be found in the impact of pet allergen exposure during the neonatal period or early childhood on IgE trajectory development, which can be modified by concomitant changes in microbial exposure (because of cesarean birth, for example) [47]. Thus, changes in the environment, via modifications induced in the gut microbiota (because of different diets, for example), could have a significant impact on the protective effect or not of early exposure to pets and thus explain the disparities found in the different studies. It is interesting to note that these studies were not carried out in the same countries: for example, the studies of Collin et al. [43, 44] and Gabet et al. [46], which yielded contradictory results, were respectively performed on children in the UK and in France, two countries with different eating habits. The role of these multiple interactions, such as exposure to allergens, intestinal microbiota and diet, need to be better understood and characterized.

### CRD-based clinical aspects

#### Allergenicity

Fel d 1 is the major allergen of domestic cats [7, 48, 49]. Anti-Fel d 1 specific IgE is found in the serum of more than 80–95% of patients allergic to cats [4, 5, 50, 51]. Crossed immunoelectrophoresis tests showed that most IgE antibodies to cat allergens in the serum of allergic patients are directed against Fel d 1, and account for 60–90% of overall allergenic activity [4, 5, 11, 50, 52]. In vivo, the allergenicity of Fel d 1 is determined by its recognition by the mannose receptor on mucosal antigen-presenting cells, such as dendritic cells or macrophages [53]. Several studies have shown that T cell response against Fel d 1 is polarized toward the Th2 pathway [54–56].

#### Positive diagnostic value

**Fig. 2:**
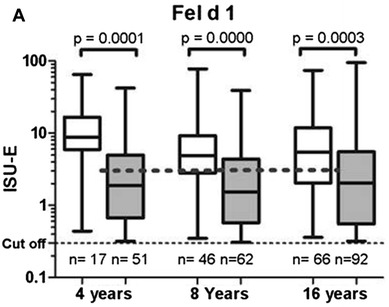
Specific IgE levels (≥ 0.3 ISU-E) to cat allergens in children with (white box plots) or without (gray box plots) symptoms to cat at 4, 8, and 16 years of age. From Asarnoj et al. [58] reprinted with permission from the publisher

The first clinical question regarding Fel d 1 is its place in the diagnostic strategy of cat allergy. Specifically, some authors question its ability to replace the overall extract in daily diagnostic practice, because theoretically, using anti-Fel d 1 specific IgE alone can lead to potential false negative results owing to the atypical profiles of sensitization with IgE directed only against other cat allergens. A very recent study tried to provide an answer to this key point. Smoldovskaya et al. [57] compared in 139 patient serum samples the results of sensitization of the whole allergen extracts in relation to the recombinant protein in biochip-based immunoassay (EIMB RAS). They reported that values for diagnostic accuracy for the cat dander extract and its major recombinant component Fel d 1 were comparable, with similar ROC curves [57]. This suggests that the global extract could be replaced by the major allergen component Fel d 1 for diagnostic purposes. Moreover, Asarnoj et al. [58], in the large BAMSE/MeDALL study, showed that testing Fel d 1 sensitization (analyzed with a chip based on ISAC Thermo Fischer technology = Mechanisms for the Development of Allergy chip) was as good as testing for IgE to cat allergen extract (ImmunoCAP) and was more predictive of cat allergy at 16 years of age (Fig. 2).

Conversely, two new studies analyzing the usefulness of CRD analysis of cat allergy in routine clinical practice drew the exactly opposite conclusion [59, 60]. In the first, native cat extract serology testing was 100% successful in detecting patients who were allergic to cats but rFel d 1 testing only 91% [59]. Thus, 9% of cat allergic patients would have not been detected with CRD testing alone. In the second, a substantial proportion (56/117; 48%) of subjects tested IgE positive for cat extracts (ImmunoCAP IgE) were negative for all the corresponding cat components (ImmunoCAP ISAC) including Fel d 1. However, as cat allergen components were not measured by unitary ImmunoCAP IgE, these results reflect probably more the lack of sensitivity of ISAC technology than of cat allergen components [60].

In another very recent study, of 70 pet allergic patients 69 had positive cat skin prick tests and 65 were sensitized to at least one feline component (Fel d 1, Fel d 2 and Fel d 4). However, the IgE against cat global extract was not tested. Of the latter 65 patients, 61 were sensitized against Fel d 1 (87.1% of the overall study group or 93.8% of patients having positive component-specific IgE), of whom 30 (46.2%) were monosensitized. Of the 65 patients, 4 were sensitized only against Fel d 2 and/or Fel d 4 (6.1%) [61].

Finally, a synthesis of the recent literature on the biological diagnosis of cat allergy shows that the data from the studies are not always consistent. The analytical performances of the anti-Fel d 1 specific IgE assay are close to those of the specific IgE assay directed against the overall extract. However, for a small number of cases with non-typical sensitization profiles (about 5–10%), the latter could have higher sensitivity. In our center, therefore, we consider that in the current state of knowledge an anti-overall extract specific IgE assay still has its place in daily practice in the positive diagnosis of cat allergy.

#### Assessment of the disease severity

**Fig. 3:**
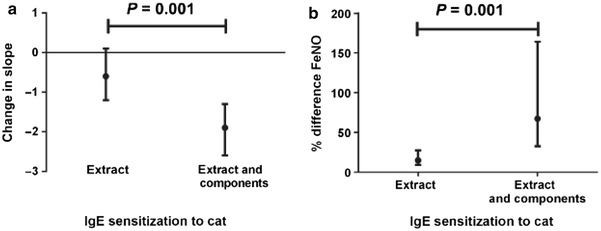
Difference in bronchial responsiveness (lower values show more responsiveness, **a**) and % difference of fraction of exhaled nitric oxide (FeNO) (**b**) between subjects sensitized to cat extract or both extract and components vs. subjects not sensitized to cat. Results are from a multiple linear regression model and adjusted for age, BMI, sex, smoking at baseline. Reference group are the subjects negative to both extract and any cat component. From Patelis et al. [60] reprinted with permission from the publisher

The correlation between the level of Fel d 1-specific IgE and the severity of symptoms was assessed in a case–control study by ImmunoCap in 140 cat-allergic children and adults from Sweden and Austria suffering from asthma and/or rhinoconjunctivitis [48]. Positive IgE response to rFel d 1 was observed in 95.6% of cat-allergic children and in 94.4% of cat-allergic adults. The IgE levels in rFel d 1 among children with asthma were significantly higher than in children with rhinoconjunctivitis and adults with asthma. Increased Fel d 1-specific IgE levels could thus be a potential risk factor for allergic asthma in children. In another recent study, IgE antibodies to Fel d 1 were also associated with current asthma and showed a strong degree of correlation (r = 0.94) with cat dander titers, which were strongly associated with the prevalence, severity, and persistence of asthma in a 19-year-old population (ImmunoCAP 250) [62]. In the study of Patelis, subjects sensitized to both cat extract and components had higher FeNO (P = 0.008) and more bronchial responsiveness (P = 0.002) than subjects sensitized only to the extract [60]. Subjects sensitized to cat components were more likely to develop asthma (P = 0.005) and rhinitis (P = 0.007) than subjects sensitized only to cat extract, which indicates the interest of CRD in cat allergy analysis, and in particular its value in testing the severity of the disease. An interesting study, comparing children with severe asthma (n = 37, age 13 years) and controlled asthmatics (n = 28, age 14 years) demonstrated that children with severe asthma had higher levels of IgE antibodies towards cat or Fel d 1 [63] (Fig. 3).

In addition, it has been shown that multi-sensitization towards more than three components including animal-derived lipocalin, kallikrein and Fel d 1 was associated with cases of severe asthma and among which occurred increased bronchial inflammation and a trend towards more courses of oral corticosteroid treatment [64]. Another study involving 696 Swedish children reported that current asthma and asthma symptoms following contact with cats were associated with co-sensitization to Fel d 1 and Fel d 4 (tested with ImmunoCAP ISAC). Asthma was associated with higher levels of component sensitization (Fel d 1 ≥ 15 ISU), and sensitization to more than one component from the same animal conferred the greatest risk [65].

Thus, when all these data are compiled, it is clear that measuring anti-Fel d 1 specific IgE levels makes it possible to better evaluate the prognosis of cat allergy. The quantitative aspect of the assay is important since the highest levels will be correlated with the most severe forms of the disease. In daily practice in our hospital, we therefore perform anti-Feld 1 specific IgE assay as soon as it is necessary to evaluate the severity of cat allergy.

### Use in specific immunotherapy

Allergen specific immunotherapy (AIT), consisting in progressive administration of increasing doses of allergens by different delivery routes (mainly subcutaneous, sublingual or oral), results in long-term allergenic desensitization [66, 67]. Designed to modify the nature of the immune response against allergens and thus the history of the disease, it is currently the only treatment whose aim is not only symptomatic but also etiological [50].

In cat allergy, AIT was initially tested with cat dander extract, which was effective in the treatment of cat allergy symptoms, particularly respiratory symptoms. For example, Alvarez-Cuesta et al. [68], tested sublingual immunotherapy with an aqueous standardized semi-purified cat dander extract in a double blind placebo-controlled study. The results obtained showed that in the active group there was a significant reduction in symptoms during the natural exposure challenge test. In addition, skin test reactivity to a standardized cat extract was better than in the placebo group [68]. However, the use of cat dander extract is constrained by different problems such as standardization, compliance and severe side effects [69].

For these reasons, and owing to its clinical predominance mentioned above, most studies now favor the use of Fel d 1 rather than global extract for cat allergy specific immunotherapy. Several molecular approaches using standardized preparations of Fel d 1 have been developed since the recombinant forms of this allergen have become available.

#### Hypoallergenic Fel d 1

To reduce the allergenicity of Fel d 1, and thus increase the security of AIT, various structural changes were made to Fel d 1. First, in order to modify the B cell epitopes, the disulfide bonds linking the Fel d 1 chains together were disrupted [70]. This leads to a decreased affinity of specific Fel d 1 IgE bound to the surface of mast cells and basophils on the FcɛRI receptor for Fel d 1. Seven candidates were thus generated and so designated hypoallergenic Fel d 1, owing to their ability to diminish IgE-binding and basophil activation [70]. In parallel, duplication of T-cell epitopes were added. Activation of T cells by these hypoallergenic Fel d 1 were thus not affected, or even increased, by this change in the structure [70]. More recently, seven recombinant mosaic proteins were generated by reassembly of non-IgE-reactive peptides of Fel d 1 which contained the sequence elements for induction of allergen-specific blocking IgG antibodies and T cell epitopes [71]. Immunization of rabbits has showed that three constructs may be useful for vaccination and induction of blocking IgG antibodies and for tolerance induction.

#### T cell epitope-containing peptides

In another approach based on the pivotal role of T cells in polarizing immune responsiveness to allergen, a team selected two peptides containing multiple T-cell epitopes from the sequence of Fel d 1. Unlike Fel d 1, these two peptides caused histamine release from basophils in < 1% of cat allergic patients and are unable to crosslink allergen-specific IgE molecules on basophils in vitro [72]. These peptides were then produced to obtain a peptide vaccine named Allervax CAT^®^, which has been tested in clinical trials. Norman et al. [72] conducted a study comparing a placebo group with three groups receiving Allervax CAT^®^ (7.5, 75 and 750 µg per dose) administered as a subcutaneous injection for 4 weeks. A high dose of Allervax CAT^®^ improved allergy symptoms after 6 weeks of treatment [72]. However, the treatment was accompanied by side effects within minutes or hours after administration [73]. Thereafter, new Fel d 1 vaccines were generated, in particular one using 12 shorter synthetic peptides, which reduced late-phase cutaneous reaction in a randomized double-blind controlled trial and late asthmatic reaction in another trial after 3–4 months of treatment [55, 74]. From a mechanistic point of view, evidence has been provided that treatment with selected epitopes from Fel d 1 resulted in suppression of both human and murine responses unrestricted to these epitopes (namely associated with suppression of responses to other epitopes within the same molecule, called linked epitope suppression), together with substantial induction of IL-10 in murine T cells that was not limited to cells specific for the treatment peptide [75].

Another product for cat peptide immunotherapy was then developed and tested in allergic rhinoconjunctivitis. This product, called Cat-PAD (Cat-peptide antigen desensitization), was the first in a new class of synthetic peptide immuno-regulatory epitopes (SPIREs). It consists of a mixture of seven small peptides derived from Fel d 1 [76]. These peptides were selected to provide a similar T cell response to that generated by cat dander in ex vivo PBMC derived from cat-allergic patients [76]. Owing to their small size (13–17 amino acids), the peptides constituting CAT-PAD cannot achieve cross-linking of IgE present on the surface of mast cells and basophils [76]. Clinical data from a series of randomized double-blind placebo-controlled studies confirm that Cat-PAD significantly reduced allergic rhinoconjunctivitis symptoms. The effects lasted for 2 years after the initiation of treatment [77, 78].

#### Recombinant fusion proteins

A third interesting approach consists in linking to Fel d 1 another molecule that may have various immunological properties in order to target both effectors of innate or adaptive immunity. For example, the fusion protein H22-Fel d 1, composed of rFel d 1 associated with a fragment of a humanized anti-CD64 antibody, has a high affinity for FcγRI, the high affinity IgG receptor, which is present on the surface of dendritic cells. In a monocyte-derived dendritic cell model, this resulted in increased uptake of Fel d 1. H22-Fel d 1 induced a semi-maturation of dendritic cells and led to a state of tolerance by promoting the secretion of cytokines such as IL-10 and IL-5 [79]. Another strategy was based on covalent linkage of Fel d 1 to carbohydrate-based particles (CBP), i.e. agarose particles [80]. The objective was to enhance the amount of Fel d 1 at the particle surface to improve phagocytosis by antigen presenting cells to subsequently induce an immunomodulatory effect on allergen-specific T cells. CBP-Fel d 1 was tested on a mouse model with cat allergy and the results obtained showed a reduction of airway inflammation and decreased levels of Fel d 1-specific IgE [81]. Zhu et al. [82] designed and tested a chimeric human-cat fusion protein composed of Fcγ1, a truncated human IgG, and Fel d 1, in a new approach to allergy immunotherapy targeting FcγRIIb, the inhibitory receptor present on the surface of mast cells and basophils. This Fcγ-Fel d 1 protein induced as expected an allergen-specific inhibition of the degranulation of both types of cell [82]. Luzar et al. [83], developed a new hypoallergenic vaccine against cat allergy using mimotopes of the major cat allergen Fel d 1 carried by bacteriophage particles. These bacteriophage constructs induced a predominant Th1 T cell response by promoting IL-2 production. There also exists a recombinant fusion protein composed of non-allergenic Fel d 1 peptides coupled with hepatitis B PreS protein [84].

**Fig. 4:**
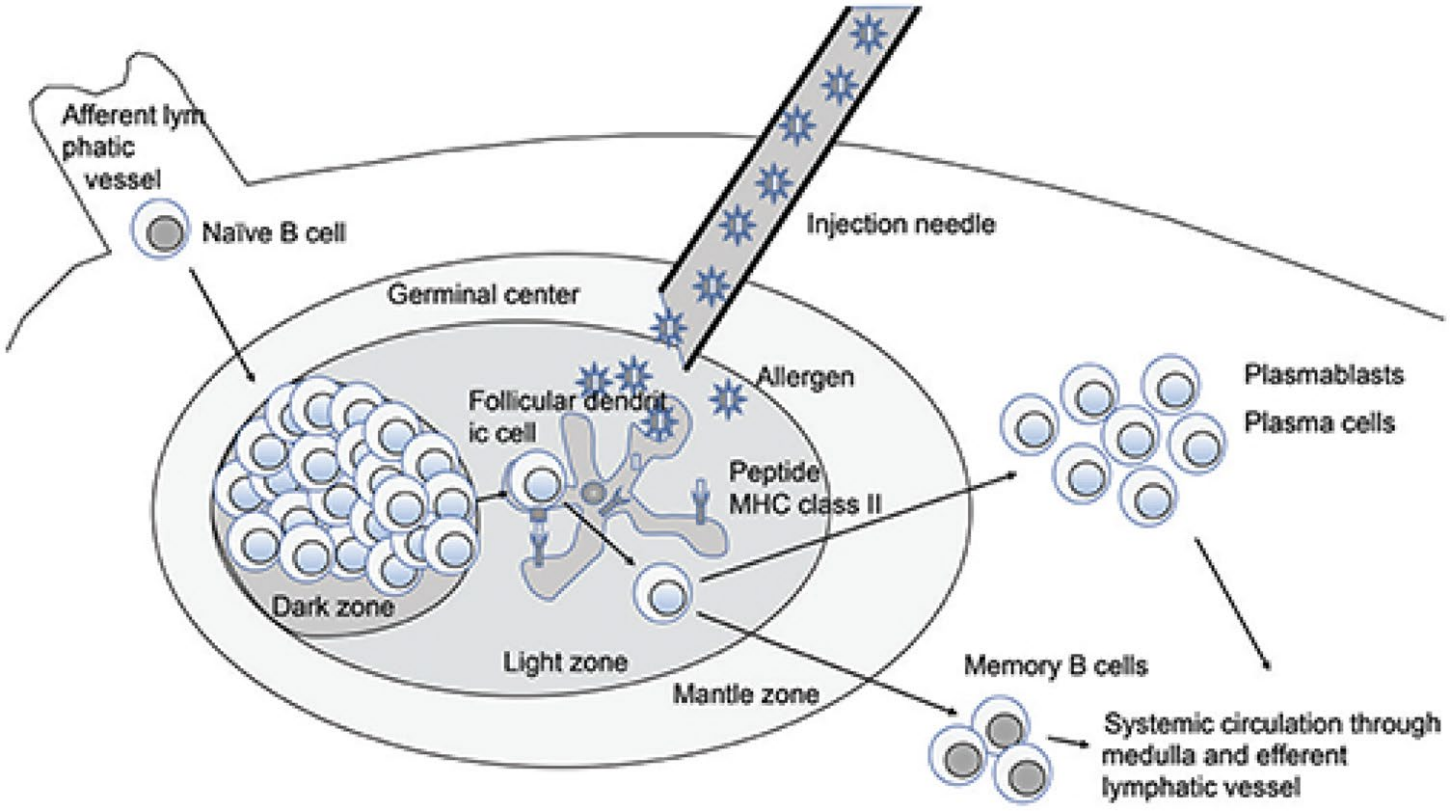
Possible mechanism of immune modulation in intralymphatic immunotherapy. From Kim et al. [87] reprints in open access

Finally, the team of Senti produced MAT-Feld 1 (modular antigen transport-Fel d 1), fusing the recombinant allergen Fel d 1 with the TAT peptide derived from the HIV virus (MAT-Feld 1) [85]. They observed a significant decrease in nasal symptoms in patients who had received three intralymphatic injections of MAT-Fel d 1 compared to those in the placebo group. In addition, MAT-Fel d 1 stimulated regulatory T cell response and increased the level of cat dander specific IgG4 [85, 86]. This raises the question of the route of administration. The form of AIT used in these latter works was intralymphatic immunotherapy (ILIT) [87] (Fig. 4). Subcutaneous immunotherapy is a lengthy process requiring many administrations over a period of 3–5 years [85] and in addition entails various side effects [88]. The sublingual route is more comfortable for the patient but the treatment has to be administered more often. Against this background, the authors argue that ILIT is an interesting alternative that warrants testing. A recent study confirmed that ILIT can rapidly improve allergy symptoms and quality of life over a period of at least a year. However, the authors reported for the first time that, in hypersensitized patients, ILIT can cause severe systemic and/or local hypersensitivity reactions (when performed with aqueous allergen extracts) [89].

## Conclusion

The appearance of the recombinant forms of Fel d 1 has led to the development of a CRD for cat allergy, which is very useful for the practitioner. Compared to the cat-specific IgE (whole extract), anti-Fel d 1 specific IgE have an equivalent or slightly lower sensitivity in terms of positive diagnosis and are correlated with disease severity and the risk of asthma occurrence. Molecular engineering has contributed to the emergence of multiple forms of Fel d 1 specific immunotherapy that are still being improved to optimize the induction of a tolerogenic immune profile. They open up great therapeutic prospects for patients in the years to come. However, it is becoming clear that the multisensitized profiles correspond to particular phenotypes of the disease, of more severe evolution. It is therefore important to carry out a complete evaluation of the cat molecular allergen, including minor fractions, to correctly characterize the patient profile, including the likely course of the disease, the potential cross-reactions and, finally, the expected immunotherapeutic response. We will deal with these aspects in the second part of this review, focusing on the less known molecular allergens of the cat, such as Fel d 2 or Fel d 4.
